# Supplementary material for: A UMLS-based spell checker for natural language processing in vaccine safety
Source: BMC Med Inform Decis Mak. 2007 Feb 12;7:3. doi: 10.1186/1472-6947-7-3 (PMC1805499; doi:10.1186/1472-6947-7-3)
Supplement: Additional file 13 — RAP application directory. Sets up directory for RAP (RDF application for PHP) source code [file 1472-6947-7-3-S13.gz › rap/doc/phpdoc/infModel/InfRule.html]

xml version="1.0" encoding="iso-8859-1"?


Docs For Class InfRule


## Class InfRule

Description

Description |
Methods (details)

This class represents a single rule in a RDFS inference model.

It primary constists of a trigger and an entailment. In the forward-chaining mode (RDFSFModel) a statement is checked, if it satisfies the trigger. If it does, a new statement is returned. In the backward-chaining mode (RDFSBModel) a find-query is checked with the entailment. If this entailment could satify the find-query, a new find-query is returned, that searches for statements that satisfy the trigger of this rule.

History:

- access: public
- author: Daniel Westphal <at d-westphal dot de mail at d-westphal dot de>
- version: V0.9.1

Located in /infModel/InfRule.php (line 27)

Method Summary

Description |
Methods (details)

boolean
checkEntailment
(object Statement $subject, mixed $predicate, mixed $object)

boolean
checkTrigger
(object Statement &$statement)

object InfStatement
entail
(object Statement &$statement)

void
getEntailment
()

array
getModifiedFind
(Node $subject, Node $predicate, Node $object)

void
getTrigger
()

void
infRule
()

void
setEntailment
(object Node $subject, object Node $predicate, object Node $object)

void
setTrigger
(object Node $subject, object Node $predicate, object Node $object)

Methods

Description |
Methods (details)

checkEntailment (line 161)

Checks, if this rule could entail a statement that matches

a find of $subject,$predicate,$object

- throws: PhpError
- access: public

boolean

checkEntailment
(object Statement $subject, mixed $predicate, mixed $object)

- object Statement
  $subject

checkTrigger (line 132)

Checks, if the statement satisfies the trigger

- throws: PhpError
- access: public

boolean

checkTrigger
(object Statement &$statement)

- object Statement
  &$statement

entail (line 193)

Returns a infered InfStatement by evaluating the statement with the entailment rule.

- throws: PhpError
- access: public

object InfStatement

entail
(object Statement &$statement)

- object Statement
  &$statement

getEntailment (line 324)

void

getEntailment
()

getModifiedFind (line 266)

Returns a find-query that matches statements, whose entailed statements would match the supplied find query.

- throws: PhpError
- access: public

array

getModifiedFind
(Node $subject, Node $predicate, Node $object)

- Node
  $subject: OR null $subject
- Node
  $predicate: OR null $predicate
- Node
  $object: OR null $object

getTrigger (line 316)

void

getTrigger
()

infRule (line 59)

Constructor

- access: public

void

infRule
()

setEntailment (line 106)

Sets the entailment of this rule The values can be NULL to match anything or be a node that has to be matched.

- throws: PhpError
- access: public

void

setEntailment
(object Node $subject, object Node $predicate, object Node $object)

- object Node
  $subject: OR NULL $subject
- object Node
  $predicate: OR NULL $predicate
- object Node
  $object: OR NULL $object

setTrigger (line 76)

Sets the trigger of this rule The values can be NULL to match anything or be a node that has to be matched.

- throws: PhpError
- access: public

void

setTrigger
(object Node $subject, object Node $predicate, object Node $object)

- object Node
  $subject: OR NULL $subject
- object Node
  $predicate: OR NULL $predicate
- object Node
  $object: OR NULL $object

Documentation generated on Thu, 7 Jul 2005 13:41:44 +0200 by phpDocumentor 1.3.0RC3
